# Control efficiency of hexaconazole-lentinan against wheat sharp eyespot and wheat crown rot and the associated effects on rhizosphere soil fungal community

**DOI:** 10.3389/fmicb.2022.1014969

**Published:** 2022-09-23

**Authors:** Xiu Yang, Zhongxiao Zhang, Yazhen Yuan, Kaiyun Wang, Yuan Chen, Hongyan Wang

**Affiliations:** ^1^Department of Plant Protection, Shandong Agricultural University, Tai’an, Shandong, China; ^2^Shandong Laboratory of Advanced Agricultural Sciences at Weifang, Peking University Institute of Advanced Agricultural Sciences, Weifang, Shandong, China

**Keywords:** seed dressing, wheat sharp eyespot, wheat crown rot, control efficiency, fungal community

## Abstract

The use of polysaccharides to induce the systemic immune response of plants for disease resistance has become an effective plant protection measure. Sharp eyespot wheat and crown rot wheat are serious diseases of wheat. In this study, the control effects of hexaconazole and lentinan (LNT) seed dressing of the two wheat diseases were evaluated by field experiments, and the effects of the seed dressing on plant growth, soil enzyme activity, and community diversity in the wheat rhizosphere were discussed. The results showed that the combined seed dressing of hexaconazole at 0.5 a.i. g·100 kg^−1^ and LNT at 4 a.i. g·100 kg^−1^ could significantly improve the control effect of the two wheat diseases. The combined treatment of hexaconazole and LNT had little effect on wheat soil enzyme activities. Different seed dressing treatments changed the fungal community structure in the wheat rhizosphere soil, and the combination of LNT and hexaconazole reduced the relative abundance of *Rhizoctonia*, *Cladosporium*, *Fusarium*, *Bipolaris*, and *Gibberella* in wheat planting soils. These findings suggested that the combined seed dressing of hexaconazole and LNT could effectively control soilborne diseases of wheat, concurrently could change in rhizosphere fungal community, and reduce in potential soilborne pathogens.

## Introduction

Wheat is an important food crop worldwide (Adrees et al., 2020). Soilborne fungal diseases cost the wheat industry billions of dollars each year (Zhang et al., 2021a). Wheat sharp eyespot is a globally prevalent soilborne disease caused by *Rhizoctonia cerealis* (Hamada et al., 2011), which can occur throughout the growth period of wheat, infecting leaf sheaths and stems and affecting the transport of nutrients and water, causing wheat lodging and dieback (Lemańczyk and Kwaśna, 2013). Wheat crown rot is an important soilborne disease caused by the infection of various pathogens such as *Fusarium pseudograminearum* and *F. graminearum*. Due to different regions, the dominant pathogens are also different (Kazan and Gardiner, 2018; Deng et al., 2020). Wheat crown rot has spread rapidly in China in the past decade, which, in severe cases, can lead to plant death, withering, and white ears (Deng et al., 2020). Soilborne diseases are usually difficult to control, and the combined infection of various soilborne fungal diseases will aggravate the occurrence of wheat diseases. In recent years, with the development of global climate change, straw turnover practices, mechanized cross-regional operations, and other labor-saving farming systems, the incidence and distribution of wheat sharp eyespot and wheat crown rot have increased significantly, seriously threatening wheat yield and quality (Hamada et al., 2011).

Although soilborne diseases can be prevented through crop rotation, selection of disease-resistant varieties, and solar disinfection, chemical control is still the most rapid and effective means when diseases break out (Wang et al., 2022). Commonly used fungicides for wheat sharp eyespot are difenoconazole and tebuconazole. However, due to the long-term single usage of chemical pesticides, *R. cerealis* has exhibited decreased sensitivity to a variety of chemical agents (Qi et al., 2014). Stem and foliar spray for wheat sharp eyespot control is time-consuming and is not environmentally friendly. Seed treatment with systemic fungicides can provide integrated control of wheat seedling and foliar diseases, improve the pesticide utilization rate, and reduce the required dosage and thus has become the focus of wheat disease management in recent years. Almasudy et al. (2015) reported that “ipconazole + metalaxyl + N-methyl-2-pyrrolidone” seed dressing treatment can significantly reduce the incidence of sharp eyespot. Ren et al. (2019) found that 6% difenoconazole-fludioxonil FSC can effectively control wheat sharp eyespot. At present, there is no registered medicine for the prevention and control of wheat crown rot in China, but many studies have shown that chemical seed dressing can effectively prevent and control the occurrence of wheat crown rot. Moya-Elizondo and Jacobsen (2016) showed that difenoconazole-mefenoxam fungicide seed treatment could reduce the severity of wheat crown rot by 29–50%, and Wang et al. (2022) reported that wheat associated with seed dressing treatment with pyraclostrobin suspension seed coating could achieve a 50.33% control effect.

Polysaccharides are complex carbohydrates with biological regulatory functions. These compounds not only provide energy for plant growth but also act as signal substances to regulate physiological processes and enhance plant disease resistance. Fungal polysaccharides have exhibited broad application prospects due to their lack of toxicity, easy degradation, environmental friendliness, and ability to induce plant disease resistance. Studies have shown that LNT can induce plant disease resistance and has an inhibitory effect on diseases. Li et al. (2013) reported that LNT showed a direct inhibitory efficiency of only 5.08% against *Phytophthora infestans* but exhibited a control efficiency of 83.00% *in vivo*. The use of polysaccharides to induce the systemic immune response of plants for disease resistance has become an effective plant protection measure.

Soilborne diseases, in addition to chemical control, can also be prevented by adjusting soil physical and chemical properties and improving soil microbial community composition. Soil microbes play key roles in ecosystems, especially fungi (Cai et al., 2021). Beneficial fungi in soil microorganisms can promote the growth and development of plants (Jia et al., 2018). Similarly, some soilborne fungi can survive in soil or diseased debris and infect plants in the form of mycelium and spores cause disease. Wheat sharp eyespot and wheat crown rot are soilborne diseases caused by pathogenic fungi, which accumulate continuously in the soil and seriously endanger the production of wheat. The complexity and diversity of microorganisms in the rhizosphere are critical for maintaining soil ecosystem homeostasis (Winter et al., 2014). Therefore, understanding the relationship between the diversity of the soil fungal community and the occurrence of wheat soilborne diseases before and after chemical seed dressing can control wheat soilborne fungal diseases by changing the nature and composition of the soil community, which provides a theoretical basis for disease control.

In a previous study, seed treatment with LNT promoted wheat germination and seedling growth in the variety Jimai 22, and the effects were dose dependent. Mechanistic analysis showed that LNT increased the transcription of genes related to alternative oxidase (AOX), β-1, 3-glucanase (GLU), and the salicylic acid signaling pathway and disease resistance, especially AOX (Zhang et al., 2017a). On this basis, we intend to systematically analyze the field control effect against two wheat diseases and the rhizosphere soil fungal community effect of LNT and hexaconazole seed treatment to provide a scientific basis for its safe and rational application.

## Materials and methods

### Test material and seed treatment

Seeds of Jimai 22 were provided by the Agricultural College, Shandong Agricultural University. Hexaconazole (95%) was provided by Shandong United Pesticide Industry Co. Ltd., China. Prepare 1 × 10^4^ mg/l with acetone and store at 4°C. The LNT with a polysaccharide content of 91% was extracted and purified in the laboratory in the early stage (Zhang et al., 2017a).

The preparation of the seed coating agent adopts the wet sand grinding ultra-fine pulverization method (Marín et al., 2016), and the other components such as the original drug, dispersant, thickener, physical stabilizer, and appropriate amount of water were mixed uniformly in a certain proportion. After pre-dispersing with a high shear emulsifier (Guangzhou Yike Laboratory Technology Co., Ltd.), the slurry was moved into a sand mill, sanded under cooling conditions, a film-forming agent was added, and a laser particle size analyzer (Jinan Runzhi Technology Co., Ltd.) after testing that the particle size of the sample is qualified, add rose essence, grind for 0.5 h, then adjust and grind the remaining water for another 0.5 h, and filter to obtain the finished product (Ren et al., 2019).

In all experiments, the coating was applied at a dose of 100 ml per 100 kg of seeds. There were 4 treatments for wheat seeds: (1) LNT (8 a.i. g·100 kg^−1^); (2) hexaconazole (1 a.i. g·100 kg^−1^) (3) (4 a.i. g·100 kg^−1^) LNT + (0.5 a.i. g·100 kg^−1^) hexaconazole, and (4) sterile water treatment (control).

### Site description and experimental design

From 2018 to 2020, a two-year field experiment was conducted on the plot of the South Campus of Shandong Agricultural University (117°13′E, 36°20′N). This area has a warm temperate semi-humid continental monsoon climate, with rain and heat in the same season, moderate rainfall, the annual average temperature is 13.2°C, and the annual average annual precipitation is 683.2 mm. Wheat is grown all year round in this area, which suffers from severe wheat sharp eyespot and wheat crown rot. It belongs to fertile brown soil. The experiment was arranged in random blocks, and each treatment was repeated 3 times. The plot area was 3.75 m^2^ and the row spacing was 0.25 m. The occurrence of wheat sharp eyespot and wheat crown rot at the overwintering, elongation, and grain filling stages was studied. All cultivation and management conditions in the experimental area were uniform. Wheat sharp eyespot disease incidences and indexes were graded from 0 to 7 as follows (Peng et al., 2014): grade 0: no disease; grade 1: the leaf sheath is affected but the stem is not affected; grade 3: the leaf sheath is affected and invades the stem, but the stem lesions are less than 1/2 of the stem; grade 5: the stem lesions are more than 1/2, but not lodging or breaking; and grade 7: lodging and withering.

Grading standard of wheat crown rot at the emergence stage and jointing stage is as follows (Erginbas-Orakci et al., 2016): grade 0: no browning symptoms on the whole plant; grade 1: browning of the coleoptile or ground stem; grade 2: plant coleoptiles and stems turn brown; grade 3: the first leaf sheath turns brown; grade 4: the second leaf sheath turns brown; and grade 5: the third leaf sheath turns brown or the whole stem turns brown and soft.

Grain-filling stage grading standard is as follows: grade 0: no browning symptoms on the innermost leaf sheath and the whole stem; grade 1: obvious browning on the innermost leaf sheath in the aerial part, and no browning and rot at the first stem node; grade 3: above ground part of the first stem node has browning and rot phenomenon; grade 5: the second stem node of the aerial part has browning and rot phenomenon; grade 7: the third stem node of the aerial part has browning and rot phenomenon; and grade 9: The lesions exceed the third stem node, with white spikes or no spikes due to disease.

Disease severity was calculated as follows (Peng et al., 2014; Zhang et al., 2017a):


$$\mathrm{Disease}\;\mathrm{severity}=\left[\begin{array}{l} \Sigma \left(\begin{array}{l} \mathrm{number}\;\mathrm{of}\;\mathrm{plants}\;\mathrm{with}a\mathrm{certain}\\ \;\mathrm{index}\;\mathrm{value}\times \mathrm{index}\;\mathrm{value} \end{array}\right)\\ /\left(\begin{array}{l} \mathrm{total}\;\mathrm{number}\;\mathrm{of}\;\mathrm{plants}\\ \;\mathrm{investigated}\times \mathrm{highest}\;\mathrm{index} \end{array}\right) \end{array}\right]\times 100\%$$

The control efficiency of each treatment was calculated as follows:


$$\mathrm{Control}\;\mathrm{efficiency}=\left[\begin{array}{l} \left(\begin{array}{l} \mathrm{disease}\;\mathrm{severity}\;\mathrm{in}\;\mathrm{control}\\ -\mathrm{disease}\;\mathrm{severity}\;\mathrm{in}\;\mathrm{treated}\;\mathrm{group} \end{array}\right)\\ /\mathrm{disease}\;\mathrm{severity}\;\mathrm{in}\;\mathrm{control} \end{array}\right]\times 100\%$$

After harvest, wheat grain number per ear and thousand seed weight were determined.

### Greenhouse emergence test and sample description

The soil used in this test was collected from the south campus of Shandong Agricultural University (117°13′E, 36°20′N) in China. The plow layer soil (5–15 cm) was collected randomly and passed through a 1-mm sieve for planting the experimental wheat. Seeds of Jimai 22 were treated as described for seed treatment. The original brown soil, brown soil treated with water only, and brown soil sown with wheat seeds treated with water only were used as controls. The plastic pots were filled with soil with uniform nutrients and particles and a water content of about 60%, a plastic pot sown with 10 treated seeds was considered a replicate, and each treatment was repeated 3 times, and cultivated in the greenhouse of Shandong Agricultural University. The growth status of crops was recorded at 7, 14, and 21 days after emergence, and the plant height among the treatments was counted. Twenty-one days after total germination in the control, the wheat rhizosphere soil was collected and divided into two parts. One subsample was stored at 4°C for the soil enzyme activity test, and the other subsample was stored in dry ice for high-throughput sequencing of fungal community diversity.

### Determination of soil enzyme activity

The activities of two soil enzymes were tested with standard methods. Soil catalase (EC 1.11.1.6, CAT) activity was determined with the KMnO_4_ titrimetric method, and urease (EC 3.5.1.5, URE) activity was determined with the colorimetric method according to Yu et al. (2019).

### DNA extraction, amplification, and sequencing

Total DNA of soil samples was extracted with the E.Z.N.A. ^®^Stool DNA Kit (D4015, Omega, Inc., United States). Primers fITS7 (5’-GTGARTCATCGAATCTTTG-3′) and ITS4 (5’-TCCTCCGCTTATTGATATGC-3′) were used to amplify the ITS2 fragment tagged with specific barcodes at the 5′ ends (Karlsson et al., 2014). The PCR mixture consisted of 25 ng of template DNA, 12.5 μl of PCR Premix, 2.5 μl of each primer, and ddH_2_O to a volume of 25 μl. The PCR conditions to amplify the eukaryotic ITS fragments consisted of an initial denaturation at 98°C for 30 s; 35 cycles of denaturation at 98°C for 10 s, annealing at 54°C/52°C for 30 s, and extension at 72°C for 45 s; and then final extension at 72°C for 10 min. After recovery and purification of PCR products by gel electrophoresis, AMPure XT beads and Qubit were used for verification and quantification, respectively. By size and quantity assessment, a library of amplicons required for subsequent sequencing was prepared. The qualified libraries were sequenced on a 250PE MiSeq platform.

### Data analysis and bioinformatics analysis

SPSS 22.0 was used for statistical analysis of the data, and Duncan’s new multiple range method was used for multiple comparisons between treatments. Paired-end reads were assigned to samples according to their barcode. After eliminating barcodes and primer sequences and merging paired-end reads, raw tags were filtered to obtain clean tags. Chimeric sequences were filtered using Verseach software (v2.3.4). For ease of analysis, sequences are usually divided into different OTUs according to a 97% similarity threshold, and each OTU is treated as a microbial species (Blaxter et al., 2005). That is to say, if the similarity is less than 97%, it can be regarded as different species, and if the similarity is less than 93% ~ 95%, it can be regarded as belonging to different genera.

Representative sequences were assigned taxonomic data with the RDP (Ribosomal Database Project) classifier. The phylogenetic relationship of different dominant OTUs in different groups was analyzed by PyNAST with the multiple sequence alignment method. OTU abundance information was normalized corresponding to the sample with the fewest sequences. Alpha diversity indexes, calculated with QIIME (Version 1.8.0), were used to represent species diversity, including Chao1, Shannon, Simpson, and observed species indices. Beta diversity analysis was used to evaluate differences of samples in species complexity. Beta diversity was calculated by principal co-ordinates analysis (PCoA) and cluster analysis by QIIME software (Version 1.8.0). Redundancy analysis (RDA) of soil colonies and soil properties using CANOCO4.5.

## Results

### Field control efficiency of hexaconazole, LNT, and their combination against wheat sharp eyespot and wheat crown rot

In the field test, seed dressing with LNT alone exhibited lower efficiency of control against wheat sharp eyespot and wheat crown rot (Tables 1, 2). During overwintering, the control effect of 8 a.i. g·100 kg^−1^ LNT on Jimai 22 was lower than 40%. Seed dressing with hexaconazole showed excellent control efficiency against two wheat diseases in the earlier stages of wheat growth. During overwintering, the control effects of hexaconazole at 1 a.i. g·100 kg^−1^ against sharp eyespot were 81.5% and 76.9%, respectively and the control effects on wheat crown rot were 75.7% and 81.4%, respectively. However, the effects decreased with time during the grain filling stage; the control effects on the two diseases were 38.8%, 35.6%, 30.5%, and 29.8%, respectively. From the field test results, the combination of LNT with a dosage of hexaconazole showed high and long-lasting control efficiency. During the wintering period, the control effects of 4 a.i. g·100 kg^−1^ LNT and 0.5 a.i. g·100 kg^−1^ hexaconazole on Jimai 22 were all above 70% and still above 40% at the grain filling stage, which were significantly higher than those of hexaconazole at 1 a.i. g·100 kg^−1^.

**Table 1:** Field effects of seed dressing agents on sharp eyespot of wheat.

Year	Treatment	Dosage (a.i. g /100 kg seed)	Control efficiency (%)			Grains per spike	Thousand seed weight(g)
			Overwintering	Elongation	Grain filling		
2018–2019	CK	-	-	-	-	37.1 ± 0.41a	42.1 ± 1.25c
	LNT	8	36.9 b	25.6c	19.8c	37.4 ± 0.32a	41.9 ± 1.76c
	Hexaconazole	1	81.5a	60.6b	38.8b	36.9 ± 0.19a	43.9 ± 1.18b
	ḤLNT	0.5·4	79.7a	66.4a	43.5a	37.9 ± 0.37a	45.9 ± 1.72a
2019–2020	CK	-	-	-	-	36.3 ± 0.38a	41.3 ± 1.33c
	LNT	8	36.3b	26.2c	20.8c	35.9 ± 0.51a	40.7 ± 1.65c
	Hexaconazole	1	76.9a	56.3b	35.6b	36.2 ± 0.22a	43.1 ± 1.22b
	ḤLNT	0.5·4	75.8a	63.3a	41.7a	36.5 ± 0.41a	45.6 ± 1.64a

CK means that seeds are treated with sterile water. H LNT stands for the combination of hexaconazole with LNT. Different lowercases in the same column indicate significantly different at *p* < 0.05 level according to Duncan’s multiple range tests.

**Table 2:** Field effects of seed dressing agents on wheat crown rot.

Year	Treatment	Dosage (a.i. g /100 kg seed)	Control efficiency (%)			Grains per spike	Thousand seed weight(g)
			Overwintering	Elongation	Grain filling		
2018–2019	CK	-	-	-	-	37.9 ± 0.25a	42.3 ± 1.06c
	LNT	8	35.5b	27.4c	20.4c	37.3 ± 0.57a	42.8 ± 1.39c
	Hexaconazole	1	75.7a	58.1b	30.5b	37.4 ± 0.26a	44.1 ± 1.27b
	ḤLNT	0.5·4	73.4a	63.3a	41.6a	37.5 ± 0.53a	45.1 ± 1.33a
2019–2020	CK	-	-	-	-	38.2 ± 0.21a	42.7 ± 1.12c
	LNT	8	35.6b	30.2c	22.7c	38.1 ± 0.14a	42.5 ± 1.24c
	Hexaconazole	1	81.4a	61.2b	29.8b	38.3 ± 0.39a	44.4 ± 1.36b
	ḤLNT	0.5·4	77.1a	72.7a	40.6a	38.2 ± 0.42a	45.4 ± 1.21a

CK means that seeds are treated with sterile water. H LNT stands for the combination of hexaconazole with LNT. Different lowercases in the same column indicate significantly different at *p* < 0.05 level according to Duncan’s multiple range tests.

The number of grains per panicle of Jimai 22 was not significantly different among the different treatments. When Jimai 22 was used in combination with 0.5 a.i. g·100 kg^−1^ of hexaconazole and 4 a.i. g·100 kg^−1^ of LNT, the thousand-kernel weights were 45.9 g, 45.6 g, 45.1 g, and 45.4 g, respectively, which represented significant increases compared with the control.

### Effects of different seed dressing treatments on the growth of wheat seedlings

**Figure 1:**
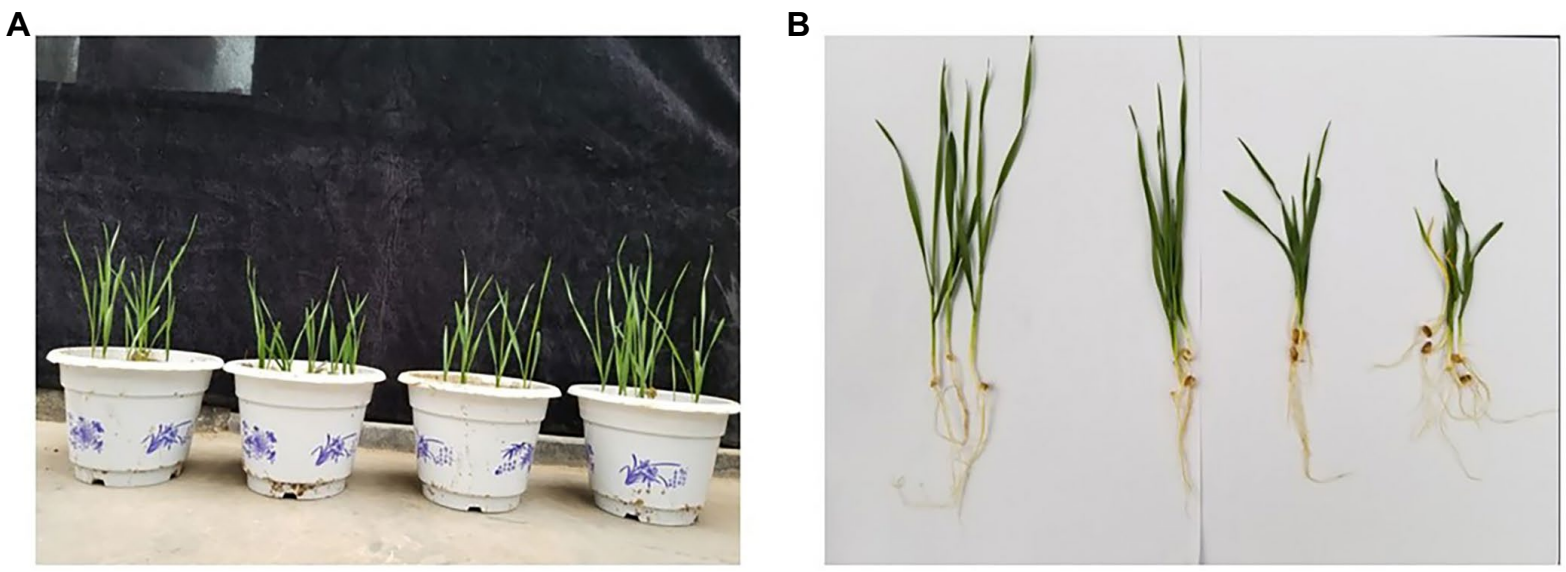
Greenhouse pot experiments on the growth of Jimai 22 with different seed dressing treatments. **(A)** Effects of different treatments on the growth of Jimai 22. Processing from left to right: CK, Hexaconazole, ḤLNT, LNT. **(B)** Effects of different seed dressing treatments on the root system of Jimai 22 The treatments from left to right are: LNT, CK, ḤLNT, Hexaconazole.

From the measured plant height data (Table 3), 8 a.i. g·100 kg^−1^ LNT seed dressing treatment promoted the growth of Jimai 22. The plant height was averaged at 7, 14, and 21 days after the seeds fully emerged, which was significantly higher than the control. Compared with other treatments, the seed plant height of the 1 a.i. g·100 kg^−1^ hexaconazole seed dressing treatment was significantly different and lower than that of other treatments. Judging from the photos of potted plants in the greenhouse and the roots of wheat seedlings (Figures 1A,B), hexaconazole has a regulating effect on the growth of wheat, showing that the root system is developed, the aerial part is short and strong, and the leaf color becomes darker, which has a certain effect of strengthening seedlings. The small dose of hexaconazole combined with LNT relieved the inhibitory effect of hexaconazole and promoted the growth of wheat seedlings.

**Table 3:** Effects of different seed dressing treatments on the growth of wheat seedlings.

Treatment	Dosage (a.i. g /100 kg seed)	Height (cm)		
		7 days	14 days	21 days
CK	-	7.16c	18.14b	26.97b
LNT	8	8.57a	21.02a	29.89a
Hexaconazole	1	6.15d	15.38c	24.25c
ḤLNT	0.5·4	7.49c	19.06b	28.16b

CK means that seeds are treated with sterile water. H LNT stands for the combination of hexaconazole with LNT. Different lowercases in the same column indicate significantly different at *p* < 0.05 level according to Duncan’s multiple range tests.

### Effect of hexaconazole and LNT seed dressing on wheat soil enzyme activity

**Figure 2:**
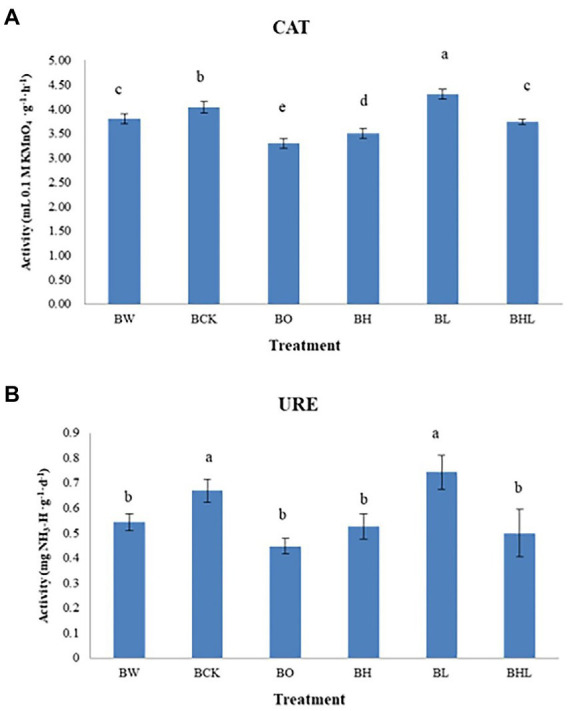
Soil enzyme activities in the different treatments. **(A)** Effects of different treatments on CAT activity in soil. **(B)** Effects of different treatments on soil URE activity. BCK – brown soil sowed with wheat seeds dressed with water only; BH – brown soil sowed with wheat seeds dressed with hexaconazole; BHL – brown soil sowed with wheat seeds dressed with hexaconazole and LNT; BL – brown soil sowed with wheat seeds dressed with LNT; BO – brown original soil; BW – brown soil with water only. Different lowercases in the same column indicate significantly different at *p* < 0.05 level.

Seed dressing treatment affected the enzymatic activity of wheat rhizosphere soil (Figure 2). Brown original soil (BO) exhibited the lowest CAT and URE activities, followed by brown soil sowed with wheat seeds dressed with hexaconazole (BH), indicating an inhibitory effect of this chemical fungicide on soil enzyme activity. Brown soil sowed with wheat seeds dressed with LNT (BL) showed the highest CAT and URE activities, followed by brown soil sowed with wheat seeds dressed with water (BCK), indicating that LNT has little effect on soil enzyme activities. The soil enzyme activities of hexaconazole and LNT treatments (BHL) were higher than those of hexaconazole-treated seeds in brown soil (BH). A moderate effect on soil enzyme activities was observed in brown soil sown with wheat seeds dressed with hexaconazole and LNT (BHL).

### Alpha diversity of the soil fungal community under by different wheat seed treatments

**Figure 3:**
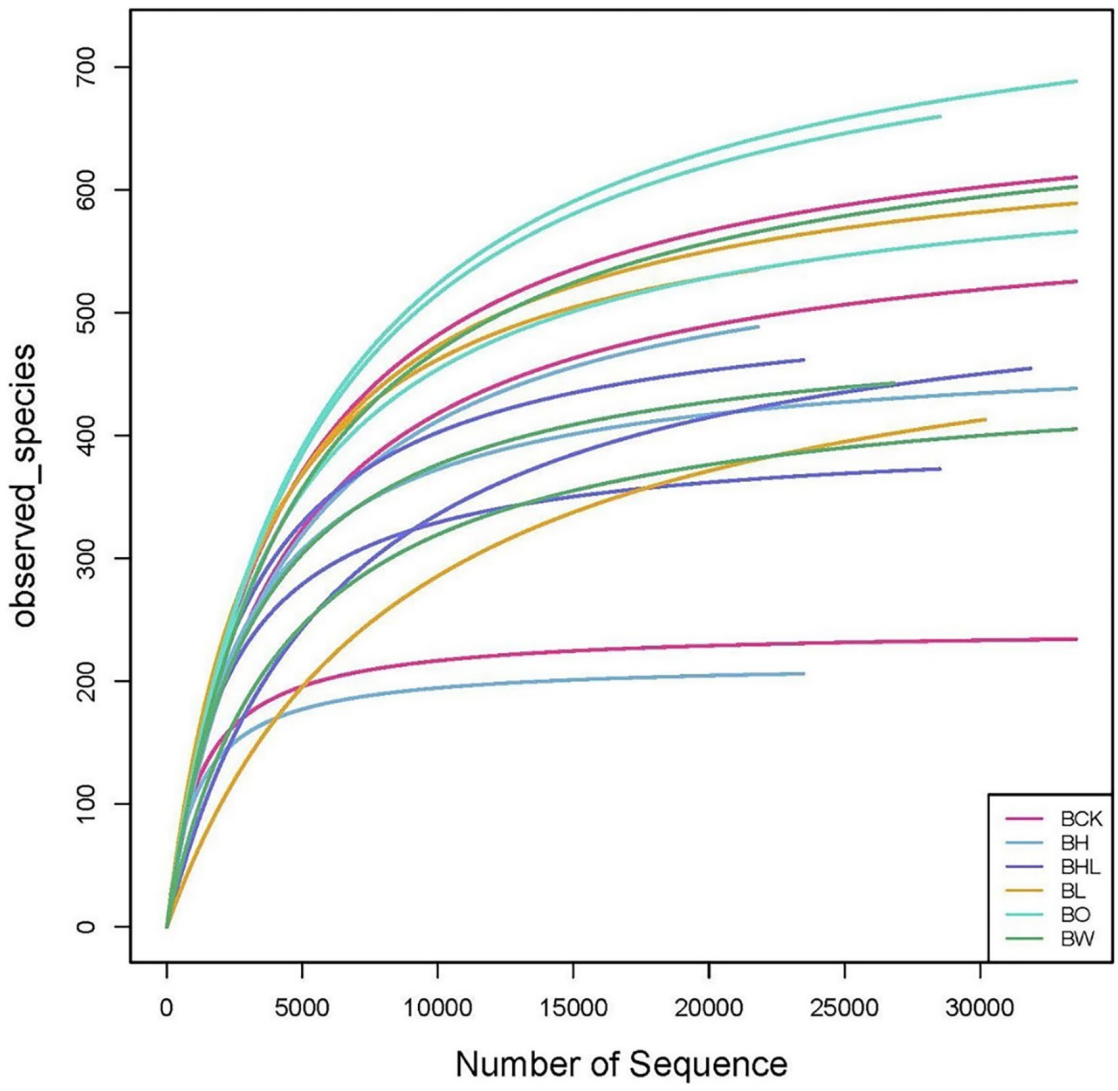
Dilution curve of soil ITS rDNA after wheat seed dressing. Divide OUT at the 97% similarity level and make a sparse curve. The curve of each sample tends to be flat, indicating that the sampling is reasonable. BCK – brown soil sowed with wheat seeds dressed with water only; BH – brown soil sowed with wheat seeds dressed with hexaconazole; BHL – brown soil sowed with wheat seeds dressed with hexaconazole and LNT; BL – brown soil sowed with wheat seeds dressed with LNT; BO – brown original soil; BW – brown soil with water only.

The dilution curve of soil can be used to assess whether the measurement is up to standard. As shown in Figure 3, with increasing sequencing data, all dilution curves increased first and then leveled off, which means that the sequencing depth was sufficient for subsequent data analysis.

As shown in Table 4, the hexaconazole-containing seed dressing treatments (BH) showed lower observed species and Chao1 indices. These values were lowest in BH, with the lowest observed species and Chao1 indices of 380.3 and 452.0, respectively, indicating the decreasing effect of hexaconazole on soil fungal number and diversity. In the LNT treatment (BL), there was no significant difference in the number of observed species and Chao1 index compared with the brown soil original soil (BO), and the relative abundance was higher. The observed species and Chao1 index of LNT and hexaconazole combined seed dressing (BHL) were 421.3 and 496.9, respectively, lower than those of the other treatments, which had a greater impact on the number and diversity of fungi in the soil.

**Table 4:** α-diversity indexes of the different treatments.

Treatment	Observed species	Shannon	Simpson	Chao1
BCK	435.0 ± 178.0ab	5.90 ± 0.93a	0.93 ± 0.07a	500.3 ± 220.2ab
BH	380.3 ± 154.4b	6.15 ± 0.26a	0.97 ± 0.01a	452.0 ± 196.0b
BHL	421.3 ± 51.6ab	5.44 ± 1.48a	0.87 ± 0.17a	496.9 ± 84.4ab
BL	504.3 ± 93.9ab	4.99 ± 1.69a	0.82 ± 0.21a	618.8 ± 42.8ab
BO	607.0 ± 52.1a	6.31 ± 0.35a	0.96 ± 0.02a	733.8 ± 97.3a
BW	466.3 ± 94.5ab	5.65 ± 1.05a	0.91 ± 0.10a	567.5 ± 100.4ab

BCK, brown soil sowed with wheat seeds dressed with water only; BH, brown soil sowed with wheat seeds dressed with hexaconazole; BHL, brown soil sowed with wheat seeds dressed with hexaconazole and LNT; BL, brown soil sowed with wheat seeds dressed with LNT; BO, brown original soil; BW, brown soil with water only. Different lowercases in the same column indicate significantly different at *p* < 0.05 level.

### **β**-Diversity of the soil fungal community in different wheat seed treatments

**Figure 4:**
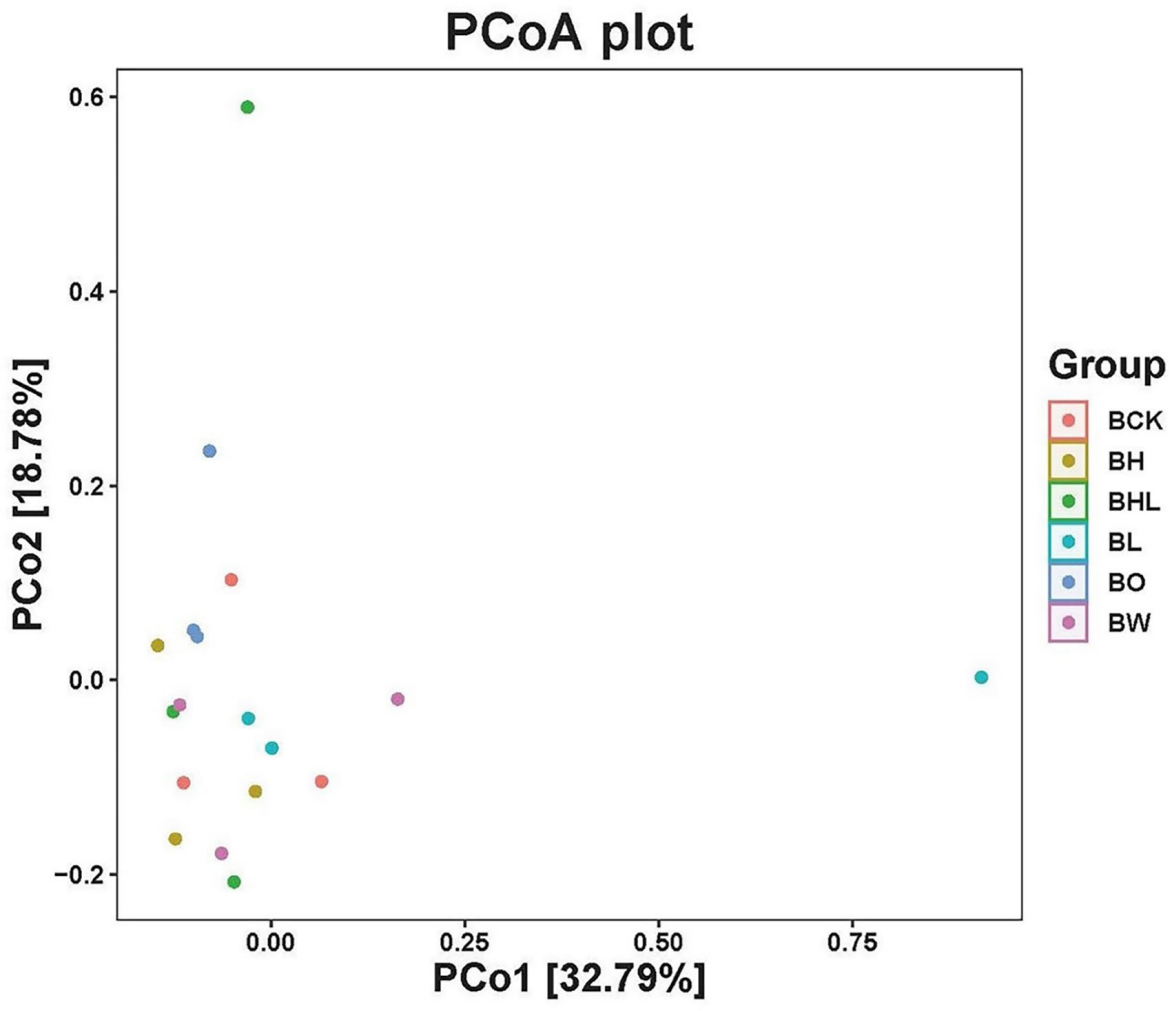
PCoA analysis of soil fungi by different seed treatments. BCK – brown soil sowed with wheat seeds dressed with water only; BH – brown soil sowed with wheat seeds dressed with hexaconazole; BHL – brown soil sowed with wheat seeds dressed with hexaconazole and LNT; BL – brown soil sowed with wheat seeds dressed with LNT; BO – brown original soil; BW – brown soil with water only.

PCoA of soil fungi in different treatments was performed based on the Weighted UniFrac distance matrix (Figure 4). The two axes explained 51.57% of the variance, the first axis PCo1 explained 32.79% of the variance, and the second axis PCo2 explained 18.78% of the variance. From the results, different wheat seed treatments changed the structure of the wheat rhizosphere soil fungal community. The distribution of BL and BHL samples was relatively discrete, and the samples were distributed farther, indicating that the LNT treatment and the combined treatment of hexaconazole and LNT had a greater impact on the soil fungal community structure.

### Effects of different seed dressing treatments on the abundance of fungal taxa in soil

**Figure 5:**
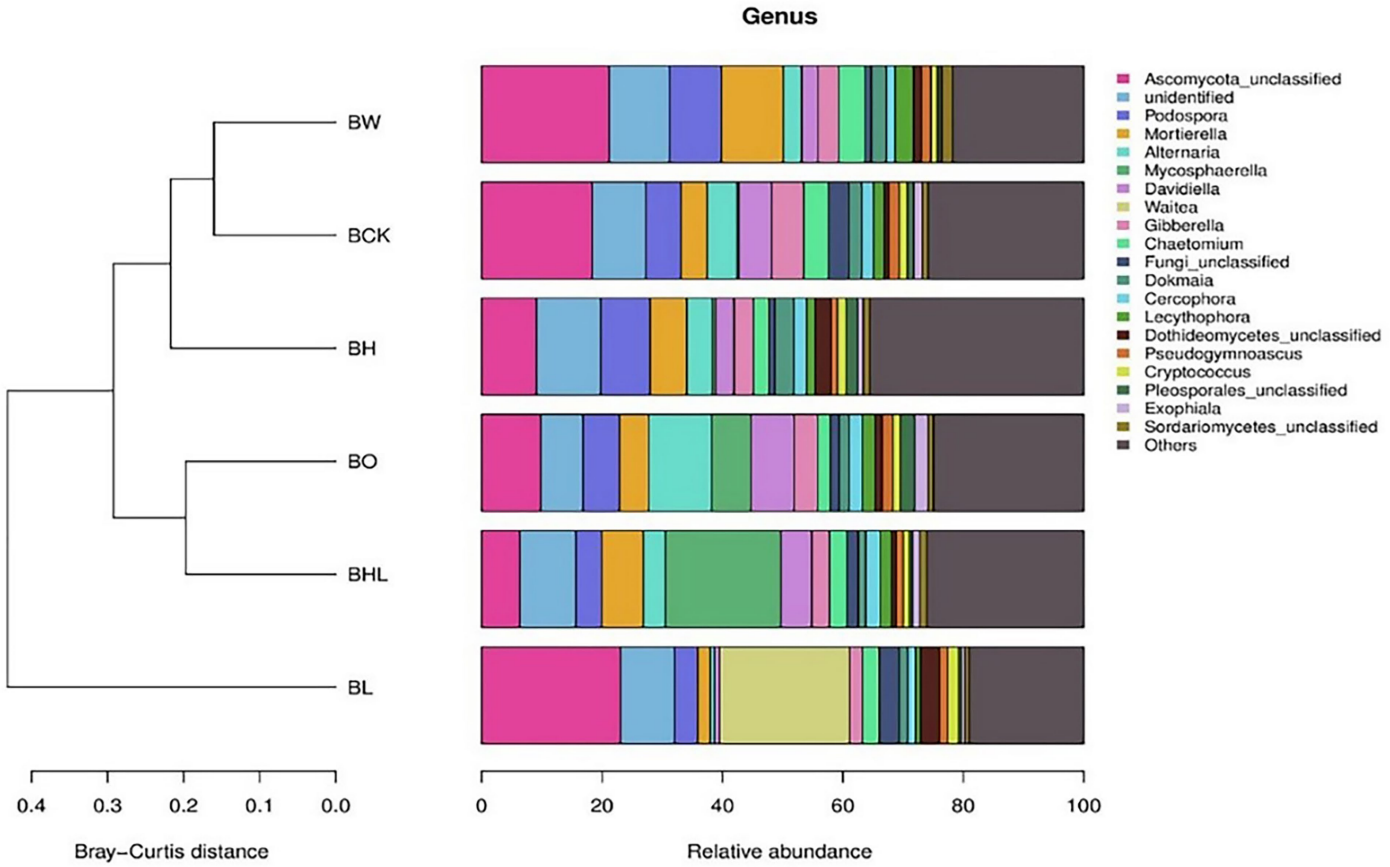
Relative abundance of fungal genera in brown soil in the different treatments. The legend shows the names of the 20 most abundant fungal genera, other genera are classified as “Other.” BCK – brown soil sowed with wheat seeds dressed with water only; BH – brown soil sowed with wheat seeds dressed with hexaconazole; BHL – brown soil sowed with wheat seeds dressed with hexaconazole and LNT; BL – brown soil sowed with wheat seeds dressed with LNT; BO – brown original soil; BW – brown soil with water only.

Figure 5 shows the similarity and species relative abundance of soil samples for different treatments. Figure 5 shows that the species compositions of BW and BCK and of BO and BHL were similar. Among the 20 most abundant species in the soil, the dominant species among the treatments was Ascomycota. Compared with the control (BCK, BO and BW), hexaconazole-containing seed dressing (BH and BHL) decreased the relative abundances of Ascomycota, *Alternaria*, and Davidiella. BHL exhibited higher relative abundances of *Mycosphaerella*, while BL exhibited higher relative abundances of *Waitea* and Dothideomycetes and lower relative abundances of *Alternaria* and *Gibberella*.

**Figure 6:**
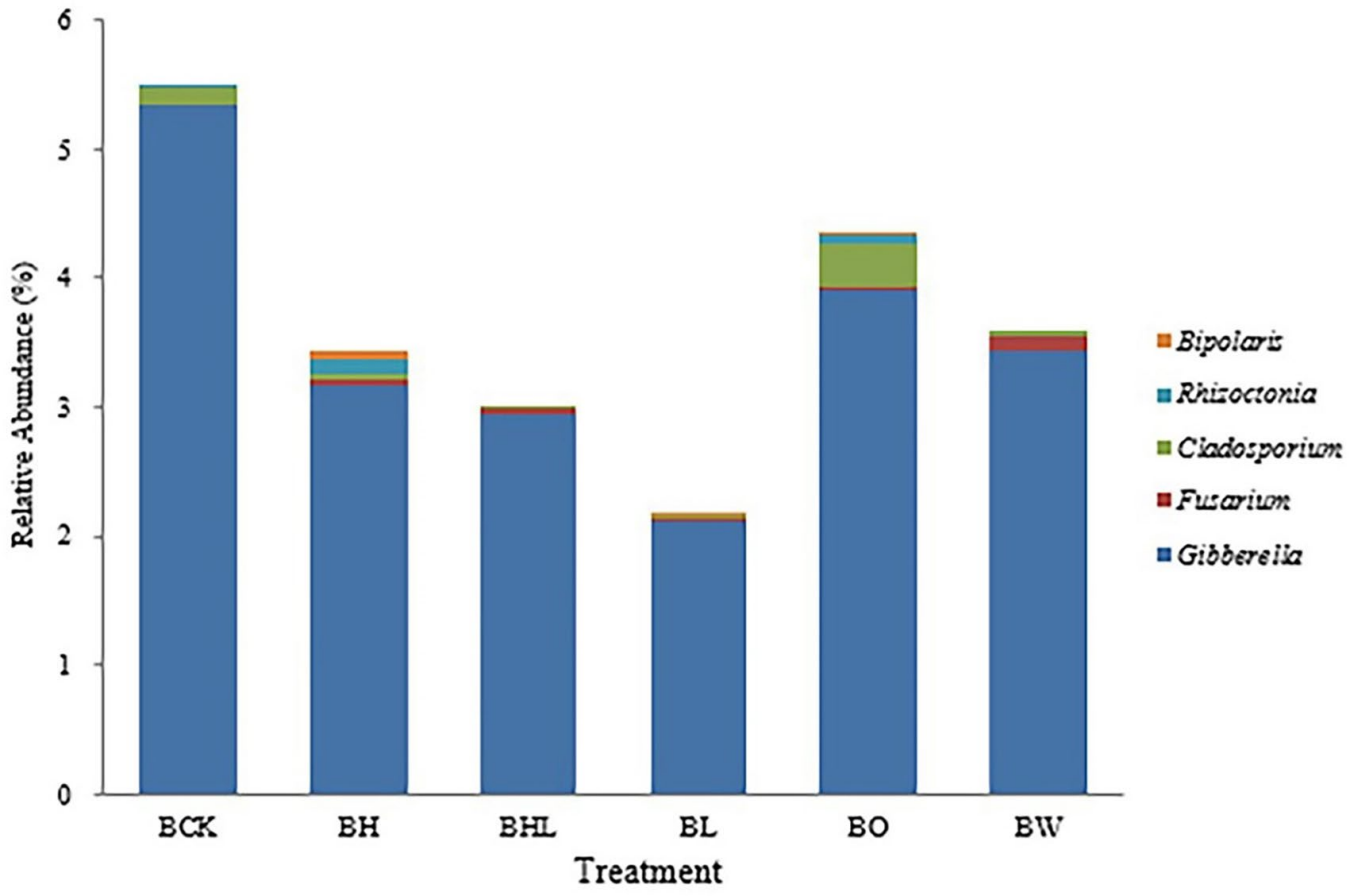
Relative abundances of the main soilborne fungi in the different treatments. Screening of relative abundances of major soilborne fungal genera in wheat at the fungal genus level. BCK – brown soil sowed with wheat seeds dressed with water only; BH – brown soil sowed with wheat seeds dressed with hexaconazole; BHL – brown soil sowed with wheat seeds dressed with hexaconazole and LNT; BL – brown soil sowed with wheat seeds dressed with LNT; BO – brown original soil; BW – brown soil with water only.

The main soilborne fungal genera of wheat were screened from the relative abundance of fungi at the genus level and made into Figure 6 according to their relative abundance. As shown in Figure 6, the relative abundances of *Bipolaris*, *Rhizoctonia*, *Cladosporium*, *Fusarium*, and *Gibberella* in BCK were 0.000%, 0.020%, 0.027%, 0.136%, 0.020%, and 5.333% respectively, close to those in BO and BW. Compared with BCK, BL showed the lowest relative abundances of the above soilborne pathogens, followed by BHL. BH exhibited higher relative abundances of *Bipolaris* and *Rhizoctonia*. Compared with BW, the relative abundance of *Fusarium* increased in BL, and the relative abundance of *Rhizoctonia* decreased. Both *Fusarium* and *Rhizoctonia* were significantly reduced in the BHL treatment compared with the BL and BW treatments.

### Effects of different wheat seed treatments on soil properties

The chemical properties of the soil by different treatments are shown in Table 5. The seen that the seed dressing treatments affected the pH, AK, AP, OM, and available N in the soil. LNT seed dressing treatment increased pH, AP, OM, and available N in soil compared with other seed dressing treatments.

**Table 5:** Chemical properties of different treated wheat soils.

Treatment	PH	AK (mg/kg)	AP (mg/kg)	OM (g/kg)	Available N (mg/kg)
BO	6.603 ± 0.003d	65.047 ± 0.839a	41.893 ± 0.577a	1.616 ± 0.032a	67.536 ± 0.467b
BW	6.603 ± 0.008d	63.780 ± 0.268ab	41.267 ± 0.228a	1.596 ± 0.026b	66.817 ± 0.137bc
BCK	6.670 ± 0.010c	57.687 ± 0.111d	39.000 ± 0.492b	1.538 ± 0.000d	63.886 ± 0.414cd
BL	6.756 ± 0.003a	63.587 ± 0.065b	40.963 ± 0.024a	1.618 ± 0.018a	72.380 ± 1.482a
BH	6.776 ± 0.013a	60.483 ± 0.405c	35.626 ± 0.003c	1.541 ± 0.012d	63.523 ± 0.771d
BHL	6.700 ± 0.000b	62.866 ± 0.389b	35.926 ± 0.044c	1.572 ± 0.012c	58.000 ± 1.663e

BCK, brown soil sowed with wheat seeds dressed with water only; BH, brown soil sowed with wheat seeds dressed with hexaconazole; BHL, brown soil sowed with wheat seeds dressed with hexaconazole and LNT; BL, brown soil sowed with wheat seeds dressed with LNT; BO, brown original soil; BW, brown soil with water only. Different lowercases in the same column indicate significantly different at *p* < 0.05 level.

**Figure 7:**
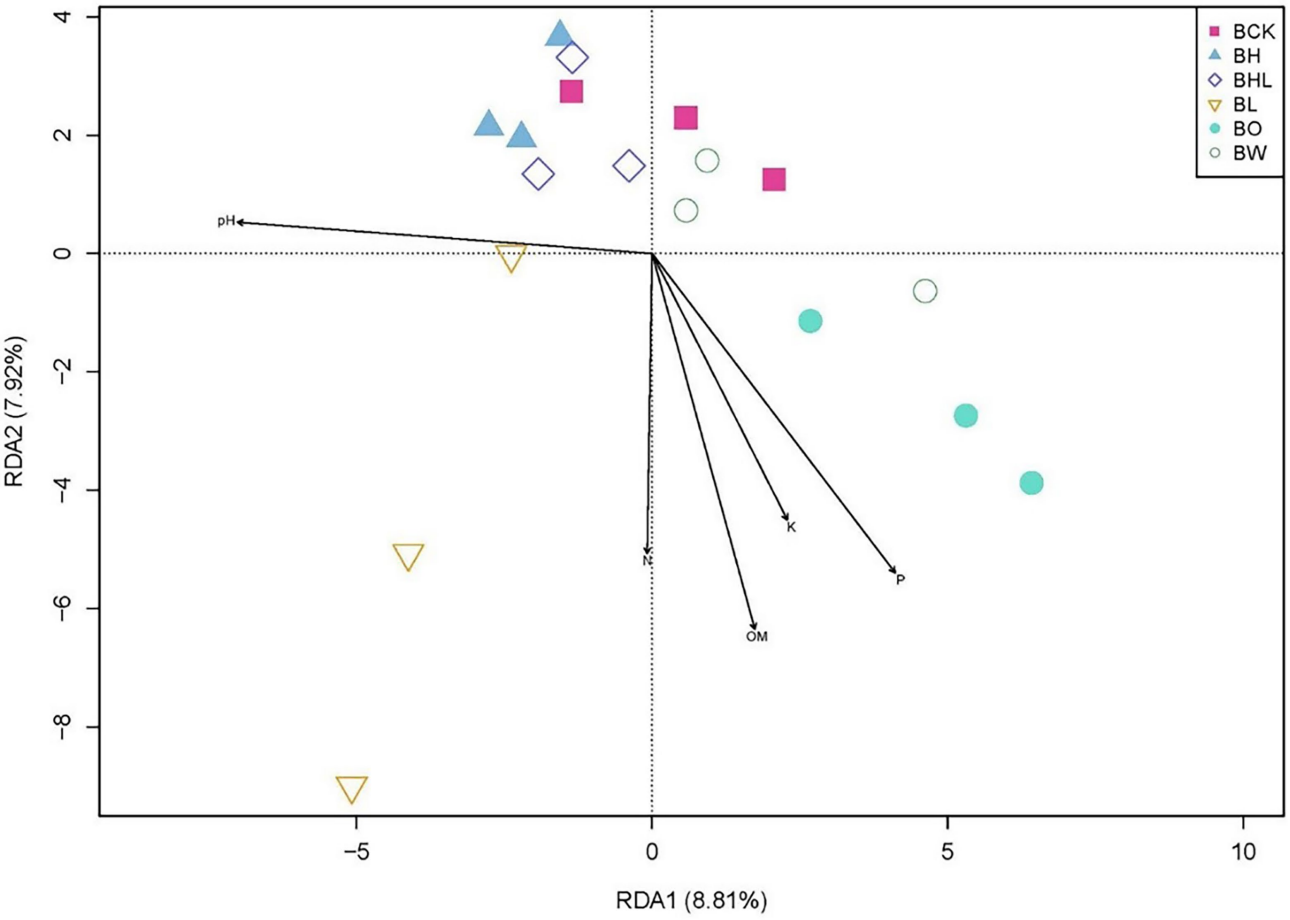
RDA analysis of soil fungal community based on seed dressing. Soil factors include P (available phosphorus), K (available potassium), N (available nitrogen), OM (organic matter), and pH. BCK – brown soil sowed with wheat seeds dressed with water only; BH – brown soil sowed with wheat seeds dressed with hexaconazole; BHL – brown soil sowed with wheat seeds dressed with hexaconazole and LNT; BL – brown soil sowed with wheat seeds dressed with LNT; BO – brown original soil; BW – brown soil with water only.

RDA explained the correlation between soil fungal communities and soil properties across treatments (Figure 7). The first axis of RDA (RDA1) explained 8.81% of the variance, and the second axis (RDA2) explained 7.92% of the variance. The closer the distance between points is, the more similar the community structure between samples. Arrows represent environmental factors, and an angle between each factor is less than 90° is positive correlation; otherwise, it is a negative correlation. AK, AP, OM, and available N were positively correlated. The length of the line connecting the arrow and the origin represents the degree of correlation between an environmental factor and the community distribution. The longer the line is, the greater the effect of the factor. The fungal community was greatly affected by pH, AP, and OM in brown soil. Moreover, the fungal communities of soil sown with wheat seeds dressed with hexaconazole-containing agents (BH and BHL) were similar to those of soil sown with non-treated wheat seeds (BCK) and with water only (BW).

## Discussion

### Effects of hexaconazole and LNT seed dressing on the growth of wheat seedlings and their control effects on two wheat diseases

Biological inducer can induce resistance to pathogenic bacteria by activating the plant’s own immune response (Walters et al., 2013). It takes a certain amount of time for biological inducer to induce plant disease resistance. Chemical control has the advantages of good effect and quick effect. The combination of fungicides and biological inducer can increase plant disease control efficiency. Baider and Cohen (2003) reported that the mixed application of mancozeb and the plant activator β-aminobutyric acid at a ratio of 1:5 had a higher control effect on cucumber downy mildew and tomato late blight than the single treatment. In addition, conventional fungicides can reduce the pathogenicity of fungi, thus making it easier for plants to induce resistance against fungi and reducing the amount and frequency of chemical fungicide applications (Oostendorp et al., 2001). Zhang et al. (2021b) found that compared with *Trichoderma asperellum* SC012 and hymexazol alone, combined application can not only reduce the dosage of chemical fungicides, but also have a better control effect on cowpea fusarium wilt.

In a previous greenhouse experiment, the combined application of LNT at 4 a.i. g·100 kg^−1^ seed and hexaconazole at 0.5 a.i. g·100 kg^−1^ seed showed a control efficiency of 75.2% against wheat sharp eyespot, which was significantly higher than that achieved with a single application (Jiang et al., 2018). From the field test results of this study, the combination of small doses of hexaconazole and LNT has a good long-term control effect on wheat sharp eyespot and wheat crown rot. In the overwintering stage, the control effect of 4 a.i. g·100 kg^−1^ LNT and 0.5 a.i. g·100 kg^−1^ hexaconazole on Jimai 22 was above 70%, and it was still above 40% in the grain filling stage, which was significantly higher than that 1 a.i. g·100 kg^−1^ of hexaconazole. The combination of a lower dosage of hexaconazole and LNT could significantly increase the wheat thousand seed weight. Seeds dressed with a combination of hexaconazole at 0.5 a.i. g·100 kg^−1^ and LNT at 4 a.i. g·100 kg^−1^ showed thousand seed weights of 45.9 g, 45.6 g, 45.1 g and 45.1 g for Jimai 22, which were significantly higher than those of the LNT single treatment. This may be because the combination of hexaconazole and LNT provides a better control effect on wheat disease during the wheat grain-filling period, which is conducive to ensuring the transport of wheat nutrients.

Studies have shown that some polysaccharides can not only regulate plant immunity, but also promote plant growth, such as chitosan can induce resistance and promote plant growth (Pichyangkura and Chadchawan, 2015). From the data of the greenhouse test, 8 a.i. g·100 kg^−1^ LNT seed dressing had a promoting effect on the growth of Jimai 22. The seed plant height of 1 a.i. g·100 kg^−1^ hexaconazole seed dressing treatment was significantly lower than that of other treatments. Hexaconazole is a triazole fungicide and has a growth-regulating effect on plants. Fletcher and Hofstra (1985) pointed out that triazolone seed dressing treatment of seeds has a significant inhibitory effect on plant growth. Domestic studies have also shown that triazole fungicides can dwarf broad bean seedlings and have the effect of strengthening seedlings (Duan et al., 2009). This is generally consistent with the greenhouse results in this study. Hexaconazole treatment has a regulatory effect on the growth of wheat. The combination of hexaconazole and LNT reduced the application dosage of hexaconazole and alleviated the inhibitory effect of high concentrations of triazole fungicides on wheat seedling growth. This is of great significance for safe wheat production.

### Effect of hexaconazole and LNT seed dressing on soil enzyme activities

Soil enzymes participate in the cycle of soil ecosystems, and their activities can be affected by many chemical and physical properties. Soil enzyme activity can directly reflect soil quality and fertility (Huang et al., 2020). Liu et al. (2020) reported that the opposite pattern of soil urease activities exhibited with heavy metal concentrations. Yu et al. (2019) reported that soil CAT activities were more sensitive to land use conversion than URE. In this study, wheat seed dressing exhibited similar effects on soil CAT and URE activities. The chemical fungicide hexaconazole showed some inhibitory effect, whereas the polysaccharide inducer LNT had little effect on soil enzyme activity. The soil enzyme activity of the hexaconazole and LNT (BHL) treatments was higher than that of the brown soil treated with hexaconazole. It may be that the combination of LNT and hexaconazole relieved the inhibitory effect of hexaconazole on soil enzyme activity, and the LNT treatment (BL) and hexaconazole (BH) treatment of wheat seeds had certain effects on wheat soil enzyme activities.

### Effects of different seed dressing treatments on the abundance of fungal taxa in soil

Soil microorganisms have received increasing attention because of their potential for optimizing plant nutrient utilization and disturbing the pathogenesis of soilborne diseases (Chang et al., 2017). Chemical fungicides applied for disease control interrupt the soil microbial activity and community structure (Fang et al., 2016). Zhang et al. (2017b) reported that repeated application of iprodione could significantly increase the relative abundance of Proteobacteria and decrease those of Chloroflexi and Acidobacteria in soil. In this study, seed dressing with LNT, hexaconazole, and their combination all influenced the fungal community diversity of wheat rhizosphere soil. Hexaconazole-containing seed dressing (BH and BHL) decreased the relative abundances of Ascomycota, *Alternaria*, and Davidiella, and BL exhibited lower relative abundances of *Alternaria* and *Gibberella*.

*Rhizoctonia*, *Cladosporium*, *Fusarium*, *Bipolaris*, and *Gibberella* are the main seed- and soilborne pathogens of wheat and can cause sharp eyespot, blight, wilt, rot, and scab diseases (Ridout and Newcombe, 2016; De Mol et al., 2018). The existence of these pathogenic bacteria creates conditions for the occurrence of wheat diseases. Cai et al. (2021) reported that fertilizer application increased the abundance of beneficial fungi in cassava rhizosphere soil and decreased the abundance of pathogenic bacteria, thereby promoting cassava growth and yield. In this study, high-throughput sequencing analysis revealed a significant inhibitory effect of the combination of LNT and hexaconazole on the relative abundances of *Rhizoctonia*, *Cladosporium*, *Fusarium*, *Bipolaris*, and *Gibberella*, changes the structure of soil fungal community, and reduces the relative abundance of pathogenic fungi. It is helpful for scientific prevention and control of wheat soilborne diseases.

### Effects of different wheat seed treatments on soil community diversity and soil properties

Microbial diversity indices are important indicators of the ecological function of soil (Wang et al., 2009). In this study, BH showed the lowest observed species and Chao1, consistent with its greater effect on soil fungal number and diversity. Hexaconazole is a triazole fungicide and is persistent in soil, but the mobility of hexaconazole is not strong. In the study by Singh (2005), hexaconazole was only moderately mobile in sandy loam soils with low organic matter content. Due to the low mobility of hexaconazole, the range of soil communities affected is also limited. Compared with other treatments, the observed species and Chao1 index of the LNT and hexaconazole combined seed dressing treatment (BHL) were lower, which had a greater impact on the number and diversity of fungi in the soil.

Soil characteristics, such as pH and nutrient content, can greatly affect the soil microbial community structure (Stefanowicz et al., 2020). Murphy et al. (2011) reported that the amount of soil organic matter is strongly linked to the size and structure of the soil microbial community. Similar to beta diversity, RDA can be used to reflect the diversity relationships among different samples. In this study, RDA showed that the soil fungal community was greatly affected by pH, available P, and organic matter.

## Conclusion

Seed treatment with LNT combined with a low dosage of hexaconazole could significantly improve the control efficiency of wheat sharp eyespot and wheat crown rot, prolong the control period, reduce the use of hexaconazole, improve the utilization rate of pesticides, and increase wheat thousand seed weight. Low-dose LNT combined with hexaconazole had little effect on the activities of soil CAT and URE. The combined seed dressing treatment (BHL) of LNT and hexaconazole had a greater effect on the number and diversity of fungi in the soil. Different seed dressing treatments changed the structure of the wheat rhizosphere soil fungal community, and the combination treatment could significantly decrease the relative abundances of the soilborne pathogens *Rhizoctonia*, *Cladosporium*, *Fusarium*, *Bipolaris*, and *Gibberella*, which reduced the incidence of soilborne diseases. In addition, the fungal community structure in wheat brown soil was related to pH, available phosphorus, and organic matter. The occurrence of soilborne diseases of crops is closely related to the physical and chemical properties of the soil. The combined seed dressing of hexaconazole and LNT can reduce the relative abundance of pathogenic fungi in soil and change the community structure of wheat rhizosphere soil fungi, which is beneficial to the prevention and control of soilborne diseases such as wheat sharp eyespot and wheat crown rot, so as to promote the yield increase of wheat.

## Data availability statement

The datasets generated for this study could be found in NCBI, BioProject: PRJNA862277.

## Author contributions

XY performed the experiments, analyzed data, and wrote the manuscript. ZZ and YY collected pathogen isolates and conducted the experiments. KW revised the manuscript. YC analyzed data and revised the manuscript. HW designed the experiments, supervised the project, and wrote the manuscript. All authors have read and agreed to the published version of the manuscript.

## Funding

This work was supported by the National Natural Science Foundation of China (32102259), the Special Fund for Agro-Scientific Research in the Public Interest of China (no. 201503130), and the Grants from Taishan Scholar Foundation of Shandong Province (tsqn202103162).

## Conflict of interest

The authors declare that the research was conducted in the absence of any commercial or financial relationships that could be construed as a potential conflict of interest.

## Publisher’s note

All claims expressed in this article are solely those of the authors and do not necessarily represent those of their affiliated organizations, or those of the publisher, the editors and the reviewers. Any product that may be evaluated in this article, or claim that may be made by its manufacturer, is not guaranteed or endorsed by the publisher.
